# Pentraxin 3 regulates neutrophil infiltration to the brain during neuroinflammation

**DOI:** 10.12688/amrcopenres.12875.1

**Published:** 2019-05-15

**Authors:** Ivana Rajkovic, Raymond Wong, Eloise Lemarchand, Rory Tinker, Stuart M. Allan, Emmanuel Pinteaux

**Affiliations:** 1 Faculty of Biology, Medicine and Health, University of Manchester, Manchester, UK

**Keywords:** Pentraxin-3, inflammation, neutrophil infiltration, neurotoxicity, neuroprotection

## Abstract

**Introduction:** The acute phase protein pentraxin 3 (PTX3) is known for its anti-inflammatory effects through downregulating neutrophil transmigration during peripheral inflammation. Furthermore, we have previously demonstrated a neuroprotective and neuroreparative effect of PTX3 after cerebral ischaemia. Here we investigated, to our knowledge for the first time, the role of PTX3 in neutrophil transmigration and neurotoxicity following lipopolysaccharide (LPS)-induced cerebral inflammation and cerebral ischaemia.

**Methods:** Neutrophil transmigration through interleukin-1β (IL-1β) activated brain endothelium and neurotoxicity of neutrophils isolated from wild-type (WT) or PTX3 knock-out (KO) mice was assessed *in vitro*. Primary cortical neuronal death after treatment with transmigrated neutrophils was quantified by lactate dehydrogenase (LDH) assay. Cerebral inflammation or ischemia was induced in WT and PTX3 KO mice via intrastriatal LPS injection or by transient middle cerebral artery occlusion (MCAo) respectively. Subsequent neutrophil infiltration in the brain was assessed by immunohistochemistry and the expression of pro-inflammatory cytokines interleukin-6 (IL-6) and IL-1β by enzyme-linked immunosorbent assay (ELISA).

**Results:** Neutrophils isolated from WT mice after intrastriatal LPS injection transmigrated significantly more through IL-1β activated brain endothelium compared to neutrophils from PTX3 KO mice. Transmigrated WT and PTX3 KO neutrophils were significantly more neurotoxic than corresponding non-transmigrated neutrophils; however, no significant differences in neurotoxicity between genotypes were observed. PTX3 reduced the number of transmigrated neutrophils to the brain after intrastriatal LPS injection. Furthermore, PTX3 KO mice showed significantly increased levels of neutrophils in the brain after LPS administration or in the ischaemic hemisphere after MCAo, compared to WT mice.

**Conclusion:** Our study shows that PTX3 regulates neutrophil transmigration in the CNS during neuroinflammation, demonstrating the potential of PTX3 as an effective therapeutic target in neuroinflammatory conditions.

## Introduction

Neuroinflammation triggered from an initial brain insult such as acute brain injury, stroke or infection is characterized by a potent central and peripheral inflammatory response that is known to activate local resident brain cells as well as peripheral immune cells, such as neutrophils, which then infiltrate into the lesion and release inflammatory mediators, exacerbating neuronal death and brain injury (Kim et al., 2016). There is extensive evidence demonstrating that neutrophils mediate disruption of the blood-brain barrier (BBB), oedema, and brain damage after ischaemic stroke, through release of reactive oxygen species (ROS), proteases and cytokines (Jickling et al., 2015). Thus, targeting neutrophils as a potential stroke treatment is an attractive therapeutic option.

Pentraxin-3 (PTX3) is an acute-phase protein known to exert anti-inflammatory and protective effects in peripheral inflammatory conditions including infection, acute myocardial infarction and lung inflammation (see (Erreni et al., 2017) for review). Indeed, PTX3 knock-out (KO) mice demonstrated greater myocardial injury, which was associated with greater neutrophil infiltration when compared to wild type (WT) mice (Salio et al., 2008). Furthermore, PTX3 has been shown to reduce neutrophil transmigration in a P-selectin-dependent manner during lung inflammation (Deban et al., 2010). Moreover, a previous published study indicated that PTX3 is stored in neutrophil granules, localised in neutrophil extracellular traps (NETs) (Jaillon et al., 2007), which could contribute to the regulation of neutrophil infiltration.

Our previous studies in stroke have demonstrated that PTX3 expressed in the brain after experimental cerebral ischemia (induced by occlusion of the middle cerebral artery (MCAo)) plays a critical role in the restoration of blood flow (Rajkovic et al., 2018) and integrity of the BBB (Rodriguez-Grande et al., 2014), and is a key regulator of long-term angiogenesis, neurogenesis and neuroprotection (Rajkovic et al., 2018). Despite these previous observations, the mechanisms by which PTX3 regulates neuroinflammation remains largely undefined, and the role of PTX3 of neutrophil transmigration in the brain during CNS inflammation remains completely unknown. This study aimed to test the hypothesis that PTX3 regulates neutrophil transmigration during neuroinflammation. Here we report, to our knowledge for the first time, that PTX3 regulates neutrophil transmigration *in vitro* and decreases neutrophil recruitment to the brain after intrastriatal lipopolysaccharide (LPS) injection and after cerebral ischaemia. In addition, neutrophil infiltration triggered neurotoxicity; however, no significant differences in neurotoxicity between genotypes was observed. Collectively, our findings suggest that PTX3 may be an effective potential therapeutic target, as it may prevent ischaemic damage through reductions in neutrophil recruitment to the brain after cerebral ischaemia.

## Methods

### Animals

Male PTX3 KO mice and WT littermates (total n=21 and n=22, respectively) were obtained by in-house breeding from heterozygote mice (bred on C57/BL6 background) (obtained from Dr. Cecilia Garlanda, Humanitas Clinical and Research Center, Rozzano, Italy), and were genotyped as described previously (Rodriguez-Grande et al., 2014). Experiments were carried out on weight-matched 12–14-week-old WT or PTX3 KO males. Animals were maintained under standard laboratory conditions: temperatures of 21±1°C, 55±10% humidity, 12 h light-dark cycle, *ad libitum* access to food and water. All animal procedures were conducted in agreement with the Animal Scientific Procedures Act (1986) and the European Council Directive 2010/63/EU, and were approved by the Home Office and Animal Welfare and Ethics Review Board, University of Manchester (UK). All experiments followed the ARRIVE (Kilkenny et al., 2012) guidelines. Animals were assigned to experimental groups in a random manner, and surgical procedures were conducted during daylight by an experimenter blinded to the genotype.

### Neutrophil isolation

Neutrophil isolation was repeated three times in three separate experiments. The 8-week-old WT and PTX3 KO mice (one WT mouse and one PTX3 KO mouse per each neutrophil isolation) were culled via cerebral dislocation. The femur and tibia were then dissected out and maintained in Roswell Park Memorial Institute (RPMI) medium (Thermo-Fisher Scientific, UK) at room temperature (RT). The bone marrow was then extracted from the femur and tibia via flushing with RPMI medium through a 25-gauge (G) needle. WT and PTX3 KO suspensions were then vigorously triturated and centrifuged at 1000 *g*, 4°C for 5 min. Subsequently, the supernatants were discarded, and 6 ml of 0.2 % saline was applied for 1 min and triturated (to lyse red blood cells), followed by 14 ml of 1.2 % saline (to restore osmolarity) and vigorous trituration. Suspensions were then passed through a 70-µM strainer and centrifuged at 2000 *g*, 4°C for 10 min. Supernatants were discarded and pellets were resuspended in 5 ml HBSS (Thermo-Fisher Scientific, UK) and triturated. Suspensions were then added to 62% isotonic percoll (Sigma-Aldrich, UK) and centrifuged at 1000 *g*, 4°C for 30 min. Excess liquid was then removed and pellets were resuspended in 10 ml HBSS and triturated. Finally, suspensions were centrifuged at 2000 *g*, 4°C for 5 min, supernatants discarded, and pellets resuspended in 5 ml RPMI.

### bEnd5 mouse brain endothelial cell line cultures

The immortalised mouse brain endothelial cell line bEnd5, which closely resembles primary brain endothelial cells, was obtained from Public Health England (Salisbury, UK). Cells were maintained in Dulbecco’s modified Eagle medium (DMEM) (Invitrogen, UK) supplemented with 10% fetal calf serum (FCS) (PAA Laboratories, UK), 1% glutamine, 1 U/ml penicillin and 100 µg/ml streptomycin (P/S) in a humidified incubator at 37°C with 5 % CO_2_. 

### Primary cortical neuronal cultures

Embryos (day 14–15 of embryonic development) were dissected out of 12–14-week-old WT pregnant C57BL/6 female mice (Charles River, UK) following euthanasia by cervical dislocation. One pregnant mouse was used for a single experiment, which was repeated three times in three separate experiments. The cortex of each embryo was dissected out and placed into starve medium (DMEM with 1% P/S) at 37°C. Trypsin (562.5 U/ml) (Sigma Aldrich, UK) and DNase (417 U/ml) (Thermo-Fisher Scientific, UK) were then added, and suspensions were placed in a shaker at 37°C for 30 min. Subsequently, FCS was added for 2 min in order to deactivate trypsin, which was then removed by three subsequent washes with wash medium (DMEM, 1% P/S, 10% FCS). Brains were then resuspended in 5 ml seeding media (Neurobasal Medium (NBM) (Thermo-Fisher Scientific, UK), 5% Plasma Derived Serum (PDS) (First Link Ltd, UK), 1% P/S, 1% glutamine (Sigma-Aldrich, UK), 2% B27 supplement with antioxidants (Thermo-Fisher Scientific, UK)) and triturated. The suspension was then passed through an 80-µM nylon filter. To prevent glial proliferation, 5-fluoro-2-deoxyuridine (30 µM, Sigma-Aldrich, UK) was added. Cells were counted and seeded at 1.8 x 10^5^ cells/cm^2^ on to Poly-D-Lysine-coated 24- or 96-well plates. On day *in vitro* (DIV) 5 medium was replaced with change medium (NBM, 5% PDS, 1% P/S, 1% glutamine, and 2% B27 supplement without antioxidants), followed by half medium change every 2 days until DIV 12-13.

### Neutrophil transmigration assay

Neutrophil transmigration was carried out as described previously (Allen et al., 2012). Briefly, 1 x 10^5^ cells/well of bEnd5 cells were seeded onto Transwell inserts (6.5 mm with 3.0 µm pore polycarbonate membrane (Sigma-Aldrich, UK)) for 24 h. bEnd5 cells were then pre-treated with recombinant mouse interleukin (IL)-1β (100 ng/ml) (R&D Systems, UK) or vehicle (low 0.1% endotoxin bovine serum albumin (BSA) in NaCl) for 4 h. Subsequently, 2 x 10^5^ neutrophils isolated from WT or PTX3 KO mice were added to the luminal (top) compartment of vehicle (WT neutrophils) or IL-1β (WT or PTX3 KO neutrophils) treated inserts and allowed to transmigrate for 24 h. Neutrophils were collected from the abluminal compartments, centrifuged at 400 *g* for 10 min and counted with a hemocytometer. Neutrophil transmigration was represented as fold increase compared to vehicle-treated cultures. 

### Neutrophil-mediated neurotoxicity

Neuronal cultures grown on to 96-well plates were incubated with WT or PTX3 KO neutrophil transmigrated conditioned media or WT or PTX3 KO non-transmigrated neutrophil conditioned media (containing 4 x 10^5^ cells/ml) for 20 h. A lactate dehydrogenase (LDH) cell death assay was then carried out to quantify neuronal cell death in cultures.

### Intrastriatal lipopolysaccharide (LPS) injection

Cerebral inflammation was achieved by intrastriatal bacterial lipopolysaccharide (LPS) injection as described previously (Giles et al., 2015). Briefly, 12–14-week-old WT or PTX3 KO male mice (five mice per experimental group for the 4-h time point; five WT and seven PTX3 KO mice for the 24-h time point) were placed in a stereotaxic frame and anaesthetised with 4% isoflurane (30% oxygen and 70% nitrous oxide gas, AbbVie Ltd, UK), followed by maintenance at 1.75%. A craniotomy was performed, and mice were injected intracerebrally using a glass microneedle with 4 μg LPS (Sigma-Aldrich, UK) or vehicle (9% NaCl) into the following co-ordinates from bregma: anterior–posterior −0.0 mm, lateral −2.0 mm, ventral −2.5 mm. Rate = 0.5 μl/min). Animals recovered for 4 h or 24 h, following which collection of cardiac blood and tissue processing was performed as described below.

### Induction of cerebral ischaemia by transient middle cerebral artery occlusion (MCAo)

12–14-week-old WT or PTX3 KO male mice (6 mice per experimental group) were subjected to experimental cerebral ischemia induced by occlusion of the middle cerebral artery (MCAo) for 15 min followed by 48 h reperfusion and recovery as described previously (Rajkovic et al., 2018). Animals that did not demonstrate a minimum of 70% decrease in CBF from pre-occlusion baseline were eliminated from the study.

### Tissues processing

Animals (n=18 PTX3 KO and n=16 WT mice) were euthanised with 4% isoflurane, and cardiac blood was removed from the right ventricle and centrifuged at 2000 *g*, 4°C for 10 min, in order to obtain plasma from blood. Mice were transcardially perfused with 0.9% saline followed by 4% paraformaldehyde (PFA) (Sigma-Aldrich, UK). Brains were then removed, post-fixed in 4% PFA for 24 h then in 30% sucrose for a further 24 h, and were serially sectioned into 30-μm sections on a freezing sledge microtome (Bright, Cambridgeshire, UK). Cryoprotectant solution (30% ethylene glycol, 20% glycerol, 0.66% sodium phosphate dibasic dehydrate, 0.079% sodium dihydrogen orthophosphate 1-hydrate, in distilled water (dH_2_O)) was used for long term storage of sections at -20°C.

### Immunohistochemistry

Free-floating brain sections were washed three times with phosphate buffered saline (PBS) and then incubated with blocking buffer (10% normal donkey serum (NDS) (Jackson laboratories, Bar Harbor, ME, USA), 0.3 % Triton X-100 (Sigma-Aldrich, UK) and PBS) for 1 h. After washing, sections were incubated overnight at 4°C with rabbit anti-mouse neutrophil primary antibody (SJC, 1:10000, a gift from Dr S. J. Campbell, University of Cambridge, UK) diluted in primary antibody buffer (2% NDS, 0.3 % Triton X-100 and PBS). Subsequently, sections were washed three times with PBS, and then incubated with Alexa-Fluor 488-conjugated secondary antibody (1:500, Donkey anti-rabbit, Cat # A-21206, RRID AB_2535792), ThermoFisher, UK) for 2 h. Following three washes with PBS, sections were mounted in dH_2_O onto glass slides, and dried in the dark at RT for 24 h. Finally, slides were cover slipped with Prolong Gold with DAPI mounting solution (ThermoFisher, UK). An Olympus BX51 upright microscope (Olympus, Japan) (10 x objective) was used to collect high power field images, which were captured via a Coolsnap ES camera (photometrics, USA) through MetaVue software (Molecular Devices, USA). Coronal sections of the same co-ordinates (approximately bregma level 0.84 mm, according to the Mouse Brain Atlas) were used for analyses of immunohistochemistry micrographs. ImageJ software (version 1.50i, National Institutes of Health, USA) was used to manually count the number of neutrophils/mm^2^ in contralateral and ipsilateral (LPS injected or ischaemic) hemispheres in three randomly selected regions in the striatum and cortex (intrastriatal LPS injection study) and penumbra (48 h stroke study) regions. All images were collected and analysed by a blinded experimenter.

### Tissue homogenates preparation

Liver and spleen were isolated and homogenised using a mechanical homogeniser (IKA, USA) in homogenisation buffer (5 μl/mg of tissue) (50 mM Tris-HCL, 150 mM NaCl, 5mM CaCl_2_, 0.02 % NaN_3_, 1% Triton X-100, protease inhibitor cocktail 1 and phosphatase inhibitors (1 mM sodium orthovanadate and 5 mM sodium fluoride)). Samples were then incubated on ice for 30 min, and then centrifuged at 14000 rpm for 30 min at 4°C, after which supernatants were stored at -80°C.

### Enzyme linked immunosorbent assay (ELISA)

An ELISA (R&D Systems, UK, Cat # DY406-05 and DY401-05) was used to detect IL-6 and IL-1β (pg/ml) levels of plasma, liver and spleen samples from WT and PTX3 KO mice (after LPS administration or MCAo) following the manufacturer’s instructions. Optical densities were read in a plate reader at 450 nm corrected for baseline at 570 nm (Synergy HT plate reader, BioTek). Concentrations of the samples were obtained by interpolating from a standard curve fitted by sigmoidal APL equation, and were expressed as pg/ml. The detection limit of the assays was 23 pg/ml for IL-6 and 16 pg/ml for IL-1β.

### Statistical analyses

All data are presented as mean values ± standard error of the mean (SEM) (n = 3-5 *in vitro* experiments; n = 5-7 *in vivo* experiments, based on previous published data (Rajkovic et al., 2018; Rodriguez-Grande et al., 2014). The normality of data was assessed with the Shapiro-wilk test, and appropriate transformations were applied when necessary. Statistical analyses performed on normally distributed data were unpaired student’s t-test (comparison of two groups), ordinary or repeated measures (where appropriate) one-way analysis of variance (ANOVA) followed by Dunnett’s corrected post-hoc analysis (comparison of multiple groups with a control group) or Bonferroni multiple comparisons post-hoc test (for comparisons between multiple groups), and repeated measures two-way ANOVA test with Sidak corrected post hoc (multiple comparisons between matched hemisphere and genotype groups). The Mann-Whitney test was used for data that were not normally distributed. P<0.05 was considered to indicate a statistically significant difference. Statistical analyses were carried out using GraphPad Prism 7.0.

## Results

### PTX3 regulates neutrophil transmigration and neuroprotection

**Figure 1.**
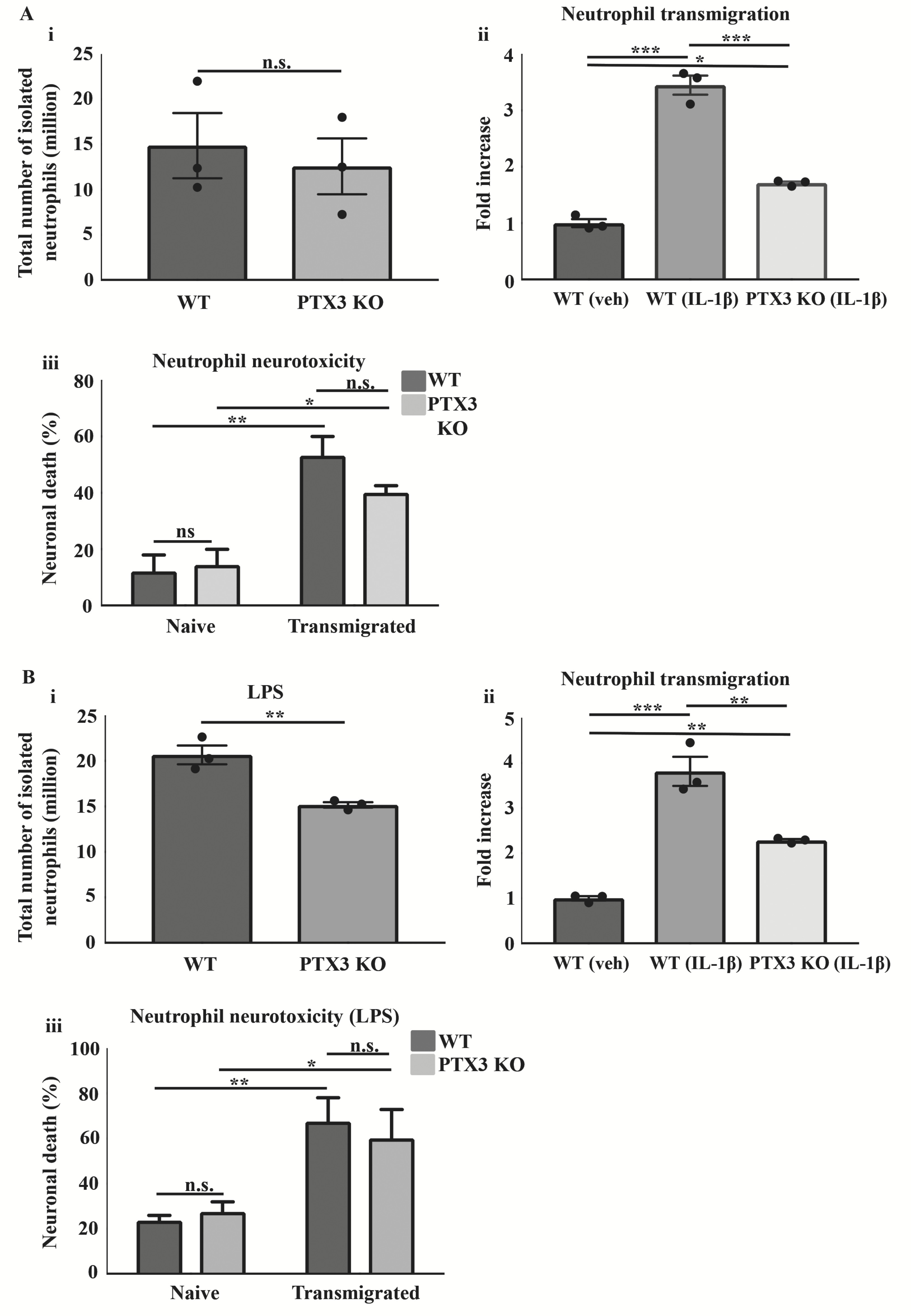
PTX3 promotes neutrophil transmigration *in vitro*. (**Ai**, **Bi**) Total number of neutrophils isolated, (**Aii**, **Bii**) neutrophil transmigration, (**Aiii**, **Biii**) and neutrophil-mediated neurotoxicity under normal and intrastriatal LPS brain injected conditions in WT and PTX3 KO mice, respectively. The total number of neutrophils and neutrophil transmigration in WT and PTX3 KO mice were quantified by cell counting with a cell haemocytometer (**Ai**, **Aii**, **Bi**, **Bii**). Neutrophil mediated neurotoxicity was quantified by LDH assay (**Aiii**, **Biii**). Statistical analyses performed using unpaired Student’s t-test (ns P > 0.05, **P < 0.01) (**A i**, **B i**), one-way ANOVA followed by Bonferroni multiple comparisons post-hoc test (*P < 0.05, **P < 0.01, ***P < 0.001) (**Aii**, **Bii**), two-way ANOVA followed by Sidak corrected post-hoc analysis (ns P > 0.05, *P < 0.05, **P < 0.01) (**Aiii**, **Biii**). All data expressed as mean ± SEM (n = 3) (**Ai–iii**, **B**).

Here we sought to investigate the role of PTX3 in regulation of neutrophil transmigration in an *in vitro* model of neutrophil transmigration through IL-1β activated brain endothelium. The number of bone marrow-derived neutrophils prepared from WT or PTX3 KO mice did not differ under normal conditions (Figure 1Ai). Neutrophils isolated from WT and PTX3 KO mice transmigrated significantly more through IL-1β activated brain endothelium, compared to WT neutrophils through vehicle treated endothelium (WT 2.4-fold, p<0.001; PTX3 KO 0.7-fold, p<0.05). However, neutrophils isolated from PTX3 KO mice transmigrated significantly less (1.74-fold, p<0.001) than WT isolated neutrophils through the IL-1β activated brain endothelium (Figure 1Aii).

We next determined the effect of WT and PTX3 KO transmigrated neutrophils on neuronal viability. Neurones cultured with WT or PTX3 KO transmigrated neutrophils for 24 h showed a significantly greater cell death (WT 3.4-fold, p<0.01; PTX3 KO 1.8-fold, p<0.05) compared to neurones exposed to naïve WT or PTX3 KO neutrophils. However, we did not find a significant difference in neurotoxicity between transmigrated WT and transmigrated PTX3 KO neutrophils (Figure 1Aiii). These data suggest that PTX3 did not affect neutrophil transmigration-induced neurotoxicity.

We repeated the study under inflammatory conditions, isolating neutrophils from WT and PTX3 KO mice after intrastriatal LPS injection (Figure 1B). Contrary to our findings under normal conditions, we noted a significant reduction (1.4-fold, p<0.01) in the number of neutrophils isolated from the bone marrow of PTX3 KO mice compared to WT mice (Figure 1Bi). Similarly, WT and PTX3 KO isolated neutrophils following LPS injection showed significantly greater transmigration through IL-1β activated brain endothelium, compared to WT neutrophils through vehicle-treated endothelium (WT 2.8–fold, p<0.001; PTX3 KO 1.3–fold, p<0.01). Furthermore, transmigration was significantly reduced (1.5-fold, p<0.01) in PTX3 KO isolated neutrophils compared to WT isolated neutrophils, through the IL-1β activated brain endothelium (Figure 1Bii). Our study also demonstrated a significant increase in percentage of neuronal death (WT 1.9-fold, p<0.01; PTX3 KO 1.2-fold, p<0.05) in cultures incubated with WT or PTX3 KO transmigrated neutrophils, compared to the corresponding naïve cultures, although no significant difference between genotypes was observed (Figure 1Biii). Collectively, these findings suggest that PTX3 is an important regulator of *in vitro* neutrophil transmigration through the brain endothelium.

### PTX3 reduces neutrophil transmigration after intrastriatal LPS injection

**Figure 2.**
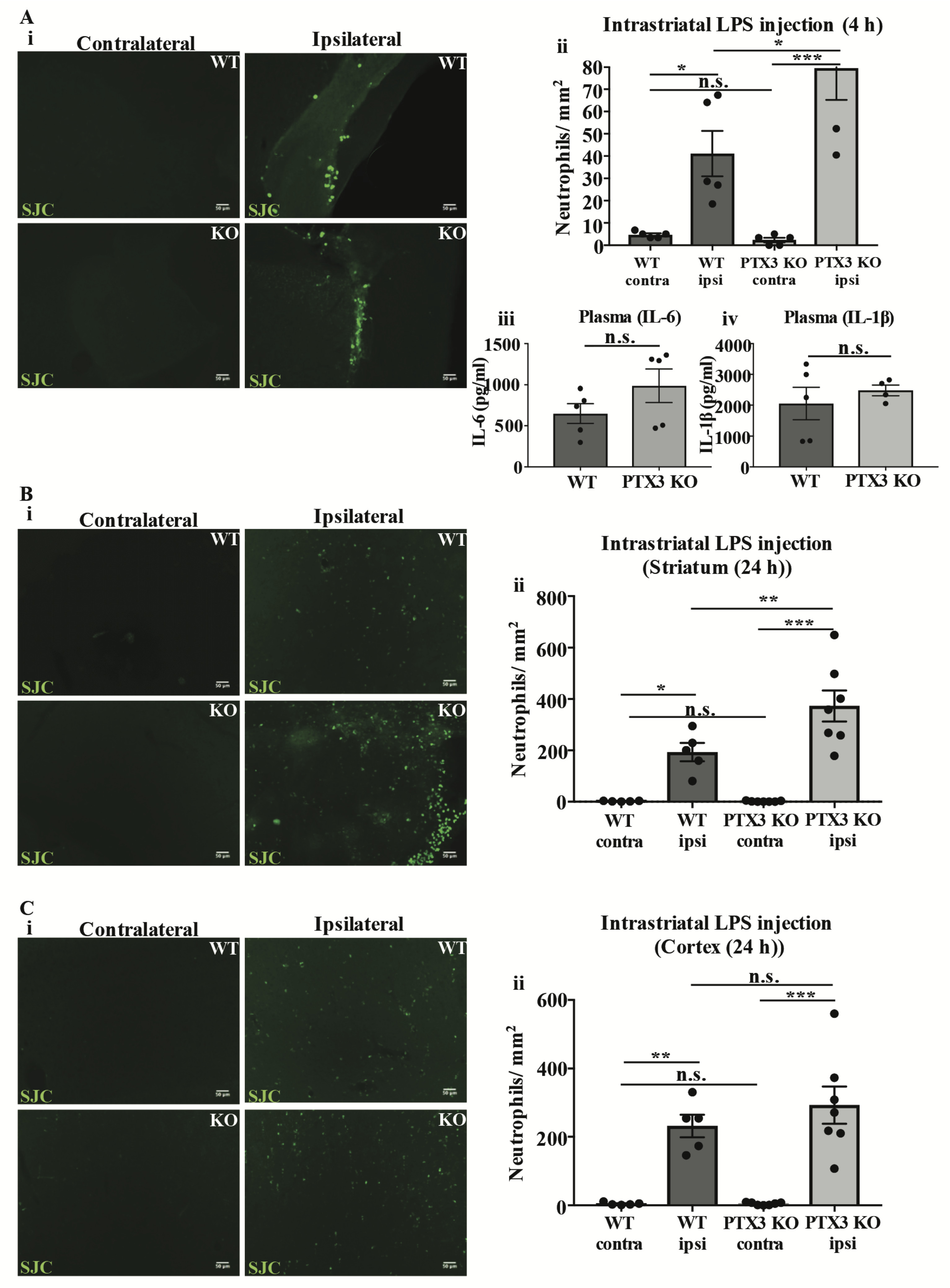
PTX3 limits neutrophil infiltration in the brain after intrastriatal LPS injection. (**Ai**, **Bi**, **Ci**) Neutrophils (green) labelled with SJC antibody in the brain of WT and PTX3 KO mice 4 h and 24 h after intrastriatal LPS injection. Scale bar, 50 μM. (**A ii**, **B ii**, **C ii**) Number of neutrophils per mm^2^ were counted manually with ImageJ software. (**A iii**, **A iv**) Levels (pg/ml) of pro-inflammatory cytokines IL-6 and IL-1β in plasma of WT and PTX3 KO mice 4 h after intrastriatal LPS injection were detected by ELISA. Statistical analyses were assessed by repeated measures two-way ANOVA followed by Sidak corrected post-hoc analysis (ns P > 0.05, *P < 0.05, **P < 0.01, ***P < 0.001) (**Aii**, **Bii**, **Cii**), or unpaired Student’s t-test (ns P > 0.05) (**Aiii**, **Aiv**). All data expressed as mean ± SEM (n = 5-7) (**A**– **C**).

Neutrophil infiltration in to the brain has been reported after intrastriatal LPS injection (Giles et al., 2015). Therefore, we examined whether PTX3 regulates neutrophil infiltration to the brain during this central inflammatory paradigm. We showed a significant increase in the number of neutrophils in ipsilateral hemispheres (LPS-injected) compared to corresponding contralateral (vehicle-injected) hemispheres in WT (7-fold, p<0.05) and PTX3 KO (33-fold, p<0.001) mice, 4 h after injection (Figure 2Ai and ii). PTX3 KO mice showed increased (2-fold, p<0.05) amounts of neutrophils in the ipsilateral hemisphere compared to WT mice. Neutrophils localised near the ventricles in both WT and PTX3 KO mice after LPS injection, and were not observed in the striatum or cortex. In addition, no significant difference in IL-6 or IL-1β levels in plasma were detected between genotypes (Figure 2Aiii and iv). We also assessed neutrophil infiltration in the striatum and cortex of WT and PTX3 KO mice 24 h after intrastriatal LPS injection. Our data show a significant increase in the number of neutrophils in ipsilateral hemispheres compared to corresponding contralateral hemispheres of WT and PTX3 KO mice, in the striatum (WT 95-fold, p<0.05; PTX3 KO 257-fold, p<0.001) and the cortex (WT 45-fold, p<0.01; PTX3 KO 63-fold, p<0.001) (Figure 2 B, C). Similar to our observation at 4 h, PTX3 KO mice showed (2-fold, p<0.01) increased numbers of neutrophils in the ipsilateral striatum compared to WT mice (Figure 2B). In contrast, we did not observe a significant difference in the number of neutrophils in the ipsilateral hemisphere between genotypes in the cortex (Figure 2C). In summary, our study demonstrates a novel role of PTX3 as a regulator of neutrophil trafficking to the brain in response to central inflammatory challenge.

### PTX3 KO mice demonstrate greater neutrophil infiltration in the brain 48 h after cerebral ischaemia

**Figure 3.**
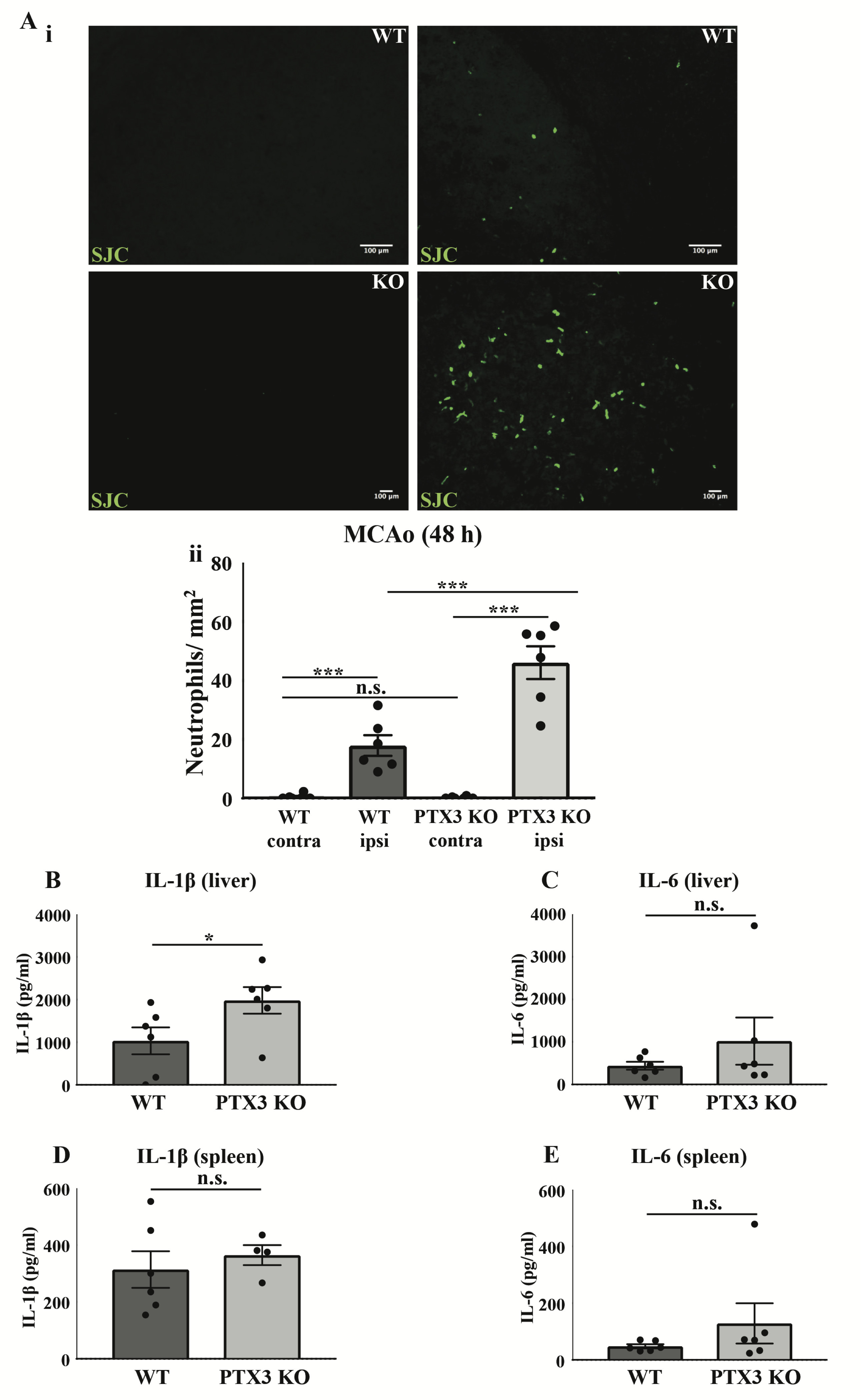
PTX3 KO mice exhibit increased neutrophil infiltration into the brain 48 h after cerebral ischaemia. (**Ai**) Neutrophils (green) labelled with SJC antibody in the contralateral and ipsilateral (ischaemic) area of the brain 48 h after stroke in WT and PTX3 KO mice. Scale bar, 100 μM. (**Aii**) The number of neutrophils per mm^2^in contralateral and ipsilateral hemispheres of WT and PTX3 KO mice were quantified manually with ImageJ software. (**B**) IL-1β and (**C**) IL-6 levels (pg/ml) in liver and (**D**) IL-1β and (**E**) IL-6 levels in the spleen of WT and PTX3 KO mice were detected by ELISA. Statistical analyses were performed with repeated measures two-way ANOVA followed by Sidak corrected post-hoc analysis (ns P > 0.05, ***P < 0.001) (**A**), and Student’s t-test (ns P > 0.05, *P <0.05) (**B**– **E**). All data expressed as mean ± SEM (n = 6 per group) (**A**– **E**).

Consequently, we examined neutrophil transmigration to the brain 48 h after cerebral ischaemia. WT and PTX3 KO mice demonstrated a significant increase (WT 38-fold, p<0.001; PTX3 KO 197-fold, p<0.001) in the number of neutrophils in the ipsilateral hemisphere compared to corresponding contralateral hemisphere (Figure 3A). Increased (2-fold, p<0.001) levels of neutrophils in the PTX3 KO ipsilateral hemisphere compared to WT mice were also observed (Figure 3A). We also assessed expression of pro-inflammatory cytokines IL-1β and IL-6 in the liver and spleen 48 h after MCAo (Figure 3 B–E). Our data show a significant increase (2-fold, p<0.05) in IL-1β expression in the liver of PTX3 KO mice compared to WT mice (Figure 3B). Levels of IL-1β and IL-6 in the plasma were below the limit of detection, and we found no significant differences between genotypes in the spleen, or the liver (IL-6) (Figure 3C, E). These findings suggest that PTX3 prevents neutrophil migration to the brain after cerebral ischaemia without affecting peripheral circulating cytokine levels.

## Discussion

The acute-phase protein PTX3 has been extensively described as a biomarker in major inflammatory disorders such as stroke, cancer or cardiovascular disease (Bonacina et al., 2013; Ryu et al., 2012; Shindo et al., 2014), and several studies indicate that PTX3 regulates neutrophil transmigration during peripheral inflammation via interaction with P-selectin (a key cell surface adhesion molecule critical for neutrophil recruitment) (Deban et al., 2010; Salio et al., 2008). However, the role of PTX3 in neutrophil infiltration during CNS inflammation remains largely unknown. Here we demonstrate for the first time that PTX3 is a key regulator of neutrophil transmigration in the CNS, but with opposing effects *in vitro* and *in vivo*. Furthermore, we demonstrate that neurotoxicity upon neutrophil transmigration is totally independent of neutrophil-derived PTX3.

Previous research has shown that PTX3 prevents excessive neutrophil infiltration, by limiting the number of infiltrated neutrophils in peripheral inflammatory conditions (Deban et al., 2010; Salio et al., 2008). First, we found that PTX3 aided neutrophil transmigration in an *in vitro* model of IL-1β activated brain endothelium, since neutrophils from PTX3 KO mice showed decreased ability to transmigrate compared to WT mice, and this effect was similar using neutrophils obtained from WT and PTX3 KO mice subjected to intrastriatal injection of LPS. However, our *in vivo* experiments are in agreement with previously published studies on cardiac ischaemia and pleural inflammation (Deban et al., 2010; Salio et al., 2008), in that PTX3 dampens down neutrophil transmigration in the brain during neuroinflammation. Indeed, we found that intrastriatal LPS injection led to a marked decrease in neutrophil infiltration to the brain, in WT mice. However, PTX3 KO mice had significantly increased neutrophil infiltration compared to WT mice, whilst levels of circulating IL-6 and IL-1β were similar between WT and PTX3 KO mice, suggesting that the differential effect of LPS on neutrophil infiltration might occur through central, rather than peripheral, inflammatory mechanisms. The inhibitory effect of PTX3 on neutrophil infiltration observed after LPS challenge was also observed after cerebral ischaemia, since PTX3 KO mice had significantly increased neutrophil infiltration compared to WT mice 48 h after MCAo. In this condition, levels of IL-1β in the spleen were also significantly increased after MCAo in PTX3 KO mice, raising the possibility that in stroke neutrophil infiltration regulated by PTX3 could involve IL-1β.

Interestingly, in our *in vivo* LPS study, infiltrated neutrophils were located near the ventricles 4 h after LPS, whereas at 24 h they were located in the striatum and cortex. Until recently neutrophils were thought to transmigrate across the BBB via microvessels into the perivascular parenchyma, and then enter the brain parenchyma (Takeshita & Ransohoff, 2012). Emerging evidence indicates that there is a population of neutrophils entering the CNS through the choroid plexus (CP), which is located in third and fourth ventricles of the brain (Meeker et al., 2012; Schwartz & Baruch, 2014). The population of neutrophils located solely by the ventricles at 4 h post LPS administration may be this population of neutrophils that have trafficked through the CP. Furthermore, we observed a significant reduction in total number of bone-marrow-isolated neutrophils from PTX3 KO mice compared to WT mice in our *in vitro* assay, which correlates with the increased number of neutrophils observed in the brains of PTX3 KO mice compared to WT mice after intrastriatal LPS injection, and may be indicative of more neutrophils trafficking from the bone marrow to the CNS in PTX3 KO mice than WT mice after inflammatory stimulation.

We have previously demonstrated that transmigrated neutrophils through an IL-1-activated brain endothelium acquired a neurotoxic phenotype *in vitro* (Allen et al., 2012), and our very recent studies found that genetic inhibition of IL-1 signalling in brain endothelial cells completely abrogated neutrophil infiltration and subsequent ischemic brain damage (Wong et al., 2019). Taking into consideration that PTX3 is readily stored in NETs (Jaillon et al., 2007), we investigated the effect of PTX3 on transmigrated neutrophil mediated neurotoxicity. Consistent with our previous study, we found that transmigrated neutrophils acquired a potent neurotoxic phenotype compared to naïve (non-transmigrated neutrophils) but found that this acquired phenotype was not significantly different between WT or PTX3 KO transmigrated neutrophils. This observation may correlate with lack of difference on acute or delayed ischemic brain damage between WT and PTX3 KO previously reported (Rodriguez-Grande et al., 2014; Rajkovic et al., 2018). Despite this, our recent study found that PTX3 mediates delayed neuroprotection after stroke, which is also supported by a previous study demonstrating that PTX3 KO mice have greater neuronal damage than WT mice in an N-methyl-D-aspartate-based epileptic seizure model (Ravizza et al., 2001). Our data taken together support the hypothesis that PTX3 is critical in the regulation of neutrophil infiltration in CNS inflammatory disease, reducing neutrophil trafficking to the brain after inflammatory insult or cerebral ischaemia, and that the neuroprotective effect of PTX3 could occur independently of PTX3-mediated neutrophil infiltration. However, our study also demonstrates that PTX3 has a completely opposite effect on neutrophil infiltration *in vitro* compared to *in vivo* inflammatory conditions, possibly involving different mechanisms due to discrepancies in the models used, further suggesting that any *in vitro* investigations must be validated *in vivo*. Our *in vivo* data, however, propose that PTX3 could be targeted therapeutically to reduce inflammatory-mediated damage and provide neuroprotection after cerebral ischaemia and potentially other cerebrovascular inflammatory diseases.

## Data availability

Figshare: Pentraxin 3 regulates neutrophil infiltration to the brain during neuroinflammation. https://doi.org/10.6084/m9.figshare.8088590 (Pinteaux, 2019).

This project contains Rajkovic_AMRC_Raw_Data.zip, which comprises underlying data stratified by the figure in which they appear.

Data are available under the terms of the Creative Commons Attribution 4.0 International license (CC-BY 4.0).

## References

[R1] Allen C Thornton P Denes A: Neutrophil cerebrovascular transmigration triggers rapid neurotoxicity through release of proteases associated with decondensed DNA. *J Immunol.* 2012;189(1):381–392. 10.4049/jimmunol.1200409 22661091 PMC3381844

[R2] Bonacina F Baragetti A Catapano AL: Long pentraxin 3: experimental and clinical relevance in cardiovascular diseases. *Mediators Inflamm.* 2013;2013:725102. 10.1155/2013/725102 23690668 PMC3649691

[R3] Deban L Russo RC Sironi M: Regulation of leukocyte recruitment by the long pentraxin PTX3. *Nat Immunol.* 2010;11(4):328–334. 10.1038/ni.1854 20208538

[R4] Erreni M Manfredi AA Garlanda C: The long pentraxin PTX3: A prototypical sensor of tissue injury and a regulator of homeostasis. *Immunol Rev.* 2017;280(1):112–125. 10.1111/imr.12570 29027216

[R5] Giles JA Greenhalgh AD Davies CL: Requirement for interleukin-1 to drive brain inflammation reveals tissue-specific mechanisms of innate immunity. *Eur J Immunol.* 2015;45(2):525–530. 10.1002/eji.201444748 25367678 PMC4357393

[R6] Jaillon S Peri G Delneste Y: The humoral pattern recognition receptor PTX3 is stored in neutrophil granules and localizes in extracellular traps. *J Exp Med.* 2007;204(4):793–804. 10.1084/jem.20061301 17389238 PMC2118544

[R7] Jickling GC Liu D Ander BP: Targeting neutrophils in ischemic stroke: translational insights from experimental studies. *J Cereb Blood Flow Metab.* 2015;35(6):888–901. 10.1038/jcbfm.2015.45 25806703 PMC4640255

[R8] Kilkenny C Browne WJ Cuthill IC: Improving bioscience research reporting: the ARRIVE guidelines for reporting animal research. *Osteoarthritis Cartilage.* 2012;20(4):256–260. 10.1016/j.joca.2012.02.010 22424462

[R9] Kim JY Park J Chang JY: Inflammation after Ischemic Stroke: The Role of Leukocytes and Glial Cells. *Exp Neurobiol.* 2016;25(5):241–251. 10.5607/en.2016.25.5.241 27790058 PMC5081470

[R10] Meeker RB Williams K Killebrew DA: Cell trafficking through the choroid plexus. *Cell Adh Migr.* 2012;6(5):390–396. 10.4161/cam.21054 22902764 PMC3496674

[R11] Pinteaux E: Pentraxin 3 regulates neutrophil infiltration to the brain during neuroinflammation. *figshare.*Dataset.2019. 10.12688/amrcopenres.12875.1 PMC6786895 31602423

[R12] Rajkovic I Wong R Lemarchand E: Pentraxin 3 promotes long-term cerebral blood flow recovery, angiogenesis, and neuronal survival after stroke. *J Mol Med (Berl).* 2018;96(12):1319–1332. 10.1007/s00109-018-1698-6 30315331 PMC6245246

[R13] Ravizza T Moneta D Bottazzi B: Dynamic induction of the long pentraxin PTX3 in the CNS after limbic seizures: evidence for a protective role in seizure-induced neurodegeneration. *Neuroscience.* 2001;105(1):43–53. 10.1016/S0306-4522(01)00177-4 11483299

[R14] Rodriguez-Grande B Swana M Nguyen L: The acute-phase protein PTX3 is an essential mediator of glial scar formation and resolution of brain edema after ischemic injury. *J Cereb Blood Flow Metab.* 2014;34(3):480–488. 10.1038/jcbfm.2013.224 24346689 PMC3948128

[R15] Ryu WS Kim CK Kim BJ: Pentraxin 3: a novel and independent prognostic marker in ischemic stroke. *Atherosclerosis.* 2012;220(2):581–586. 10.1016/j.atherosclerosis.2011.11.036 22178425

[R16] Salio M Chimenti S De Angelis N: Cardioprotective function of the long pentraxin PTX3 in acute myocardial infarction. *Circulation.* 2008;117(8):1055–1064. 10.1161/CIRCULATIONAHA.107.749234 18268142

[R17] Schwartz M Baruch K: The resolution of neuroinflammation in neurodegeneration: leukocyte recruitment via the choroid plexus. *EMBO J.* 2014;33(1):7–22. 10.1002/embj.201386609 24357543 PMC3990679

[R18] Shindo A Tanemura H Yata K: Inflammatory biomarkers in atherosclerosis: pentraxin 3 can become a novel marker of plaque vulnerability. *PLoS One.* 2014;9(6):e100045. 10.1371/journal.pone.0100045 24936646 PMC4061039

[R19] Takeshita Y Ransohoff RM: Inflammatory cell trafficking across the blood-brain barrier: chemokine regulation and *in vitro* models. *Immunol Rev.* 2012;248(1):228–239. 10.1111/j.1600-065X.2012.01127.x 22725965 PMC3383666

[R20] Wong R Lenart N Hill L: Interleukin-1 mediates ischaemic brain injury via distinct actions on endothelial cells and cholinergic neurons. *Brain Behav Immun.* 2019;76:126–138. 10.1016/j.bbi.2018.11.012 30453020 PMC6363965

