# Supplementary material for: Pentraxin 3 regulates neutrophil infiltration to the brain during neuroinflammation
Source: AMRC Open Res. Author manuscript; Available in PMC 2019 Oct 10. (PMC6786895; doi:10.12688/amrcopenres.12875.1)
Supplement: Peer review history

# Peer review: Reviewer response for version 1

**DOI:** 10.21956/amrcopenres.13941.r26420

**Published:** 2019-07-02

**Authors:** Sébastien Jaillon

**Affiliations:** 1 Humanitas Clinical and Research Center, Humanitas University, Milan, Italy

In this manuscript, Rajkovic et al aimed to assess the role of PTX3 in neutrophil infiltration to the brain. 

 Authors showed increased recruitment of neutrophil in the brain of PTX3 KO mice, compared to wild type animals. The study is interesting and the manuscript well written. 

 However, the study has some limitations.

 In particular, the discrepancy observed between *in vitro* and *in vivo* experiments remains unexplained.

 Some specific questions:

 Is it possible to modify the transmigration of neutrophils adding recombinant PTX3 in the system? 

 The authors did not take into account the production of PTX3 by the bEnd5 cells. Why? When stimulated with IL-1b, endothelial cells can produce PTX3. 

 In their *in vivo* models, authors should assess whether the levels of chemokines involved in recruitment of neutrophils are altered in PTX3 ko mice. 

 Is it possible to limit neutrophil infiltration in the brain by administrating recombinant PTX3?

I confirm that I have read this submission and believe that I have an appropriate level of expertise to confirm that it is of an acceptable scientific standard.

# Peer review: Reviewer response for version 1

**DOI:** 10.21956/amrcopenres.13941.r26391

**Published:** 2019-05-29

**Authors:** Giamal N. Luheshi, Argel Aguilar Valles

**Affiliations:** 1 Department of Psychiatry, Douglas Mental Health University Institute, McGill University, Montreal, QC, Canada; 2 Department of Neuroscience, Carleton University, Ottawa, Canada

The manuscript by Rajkovic et al investigated the role of Pentraxin 3 (PTX3) on neutrophil transmigration to the brain using  *in vitro *and  *in vivo *models. This is a very interesting study that builds on the authors substantial and innovative work on the role of neutrophils in the regulation of neuroinflammation, particularly in stroke models. The focus on PTX3 is novel and by investigating the role of this acute phase protein the authors hope to identify a suitable target for therapeutic intervention. The manuscript is very well written with very detailed methods and results section and the outcomes were discussed in the context of the wider field. There are a number of relatively minor  *general *and more  *specific *comments that the authors may want to address to further clarifying some aspects of the work:

1. Whilst the use of intracerebral injection of LPS is somewhat justified as a model of neuroinflammation, it is rather severe and importantly of little translational value, compared to the MCAO approach and even to systemic LPS which has been shown previously to trigger significant neutrophil transmigration through an IL-1b dependent manner. 
2. It is surprising, given the history of this group, that little attention was paid to central cytokines as opposed to systemic ones measured in the  *in vivo *studies of the present investigation. Particularly important would have been assessing the levels of not just the pro-inflammatory mediators but also the anti-inflammatory ones e.g.IL-1ra (in which the authors have vast experience) and IL-10. Also, interesting would have been assessing the level some of the chemoattractant chemokines involved in the neutrophil transmigration process, especially in the KO animals. It would have been interesting to see if the levels of these mediators changed in the brain in line with the increased transmigration process that the authors reported in the KO animals.
3. The finding that the mechanism underlying the regulation of neutrophil infiltration by PTX3 is possibly independent from its reported neuroprotective effects is interesting. Did the authors consider testing this in neutropenic animals? 
4. The  *In vitro *and  *in vivo *results are very clear, yet discrepant. Given the strength of the effects in the two systems, I believe is worth going further in the analysis of this discrepancy. For example:

- Is it possible that survival of PTX3 KO neutrophils is compromised in a wildtype milieu? 
- Are PTX3 KO endothelial cells expressing something that promotes transmigration (which is obviously not present in the  *in vitro *system)? 
- What would happen to WT or PTX3KO neutrophil transmigrating through PTX3 KO endothelial cells? 

It is understandable why the authors used the  *in vitro *approach to investigate their hypothesis, however given the different results in the two systems this rather distracts from the most interesting findings *in vivo. *I think a stronger justification for including these results and more in-depth discussion, other than stating *…possibly involving different mechanism..*is warranted here.

We confirm that we have read this submission and believe that we have an appropriate level of expertise to confirm that it is of an acceptable scientific standard.
